# Fracture and Fatigue Assessment of Bonded Composite Patch Repairs in Notched and Cracked Plates

**DOI:** 10.3390/polym18080912

**Published:** 2026-04-08

**Authors:** Bertan Beylergil, Hasan Ulus, Mehmet Emin Çetin, Halil Burak Kaybal, Sefa Yildirim, Abdulrahman Al-Nadhari, Mehmet Yildiz

**Affiliations:** 1 Department of Mechanical Engineering, Alanya Alaaddin Keykubat University, Alanya, Antalya 07450, Turkey; 2 Composite Technologies Center of Excellence, Istanbul Technology Development Zone, Sabanci University, Pendik, Istanbul 34906, Turkey; 3 Faculty of Engineering and Natural Sciences, Sabanci University, Tuzla, Istanbul 34956, Turkey; 4 Huglu Vocational School, Selcuk University, Konya 42710, Turkey; 5 Aeronautical Engineering Department, Necmettin Erbakan University, Konya 42090, Turkey; 6 Department of Industrial Design, Faculty of Fine Arts, Kirikkale University, Kirikkale 71450, Turkey

**Keywords:** crack–notch interaction, stress-intensity factor, composite patch repair, repair efficiency, fatigue life

## Abstract

This study presents a unified mechanics-based framework for evaluating bonded composite patch repairs. Discrete fracture, fatigue, and adhesive responses are transformed into continuous master equations over the design space. Low-order polynomial surfaces model stress intensity and concentration responses, enabling continuous prediction of repair performance without repeated finite-element analyses. A fracture-based repair efficiency index is derived from the analytical master surface. This index quantifies the average reduction in crack-driving force across the domain. Combined with adhesive stiffness and strength, it defines an adhesive-based repair efficiency index (A-REI), providing a direct link between structural response and material properties. The results show that repair effectiveness is strongly influenced by both geometric severity and adhesive properties. Fatigue performance decreases significantly with increasing notch ratio in single-sided repairs. Double-sided configurations maintain consistently higher efficiency. Symmetric reinforcement more effectively reduces stress concentration, with improvements exceeding 40% at intermediate notch ratios. Adhesive selection is governed by stiffness and strength. Structural adhesives achieve significantly higher A-REI values, whereas compliant adhesives contribute negligibly. Overall, repair symmetry controls the magnitude of improvement, while adhesive properties determine performance ranking. This framework provides a clear, practical basis for design and material selection.

## 1. Introduction

Structural components containing notches or cracks are more susceptible to accelerated damage accumulation because geometric discontinuities amplify local stresses and fracture-driving forces. In aerospace, marine, and civil engineering applications, where metallic plates frequently operate near their structural limits, such defects can substantially reduce residual strength and fatigue life if left unrepaired. Adhesively bonded composite patches, particularly carbon fiber-reinforced polymer (CFRP) systems, have therefore emerged as effective lightweight repair solutions for extending the service life of cracked metallic structures. These patches redistribute applied loads away from the crack tip, thereby reducing stress intensity factors (SIFs) and slowing fatigue crack growth. Experimental studies have demonstrated fatigue-life improvements exceeding 2.5 times compared to unrepaired specimens [1]. Furthermore, prestressed CFRP patches enhance repair effectiveness by reducing the mode I SIF from 8.3 MPa√m to as low as 2.1 MPa√m, which helps prevent crack opening and significantly retards crack growth [2].

In addition to flat plates, more complex structural configurations such as cylindrical shells and pipes, which are commonly used in pressure vessels and offshore systems, have also benefited from bonded composite repairs. Zarrinzadeh et al. reported reductions exceeding 60% in fatigue crack growth rates in pressurized aluminum pipes reinforced with CFRP patches and emphasized that patch asymmetry can alter crack propagation paths [3]. Similarly, Coelho et al. investigated single- and double-patch configurations subjected to repeated low-velocity impact loads until perforation. Their findings indicated that double-patch repairs provided superior performance, with a 97.1% increase in maximum load capacity and a 51.2% increase in stored elastic energy under a 6 J impact, while also reducing maximum displacement by approximately 50% compared to single-patch configurations. Impact bending stiffness was identified as a reliable metric for monitoring progressive damage in these repaired structures [4].

The mechanical interaction between cracks and geometric discontinuities, such as holes or notches, is critical in determining local stress fields and crack-driving forces. Analytical studies have demonstrated that stress amplification or shielding effects are highly dependent on the size, proximity, and relative orientation of cracks and holes [5]. However, most existing research examines cracks and holes independently, leaving their combined effects in bonded composite repairs insufficiently explored. Under repeated impact loading, particularly in marine and offshore environments, double-patch repair configurations have shown markedly improved damage tolerance [6].

From a modeling standpoint, fracture-mechanics–based fatigue-life predictions for cracked aluminum panels repaired with bonded composite patches have demonstrated strong agreement with experimental results. These investigations reveal that adhesive behavior, particularly interfacial disbonding and adhesive stiffness, exerts a greater influence on crack growth than patch thickness alone. Mindlin plate formulations, validated by three-dimensional finite element simulations, have enabled accurate estimation of stress intensity factors and their correlation with experimentally measured crack growth rates up to large crack lengths, beyond which adhesive debonding becomes increasingly significant [7]. In more demanding environments, such as submerged tubular joints, bonded composite repairs have achieved fatigue-life extensions of up to five times, although repair effectiveness is highly dependent on joint geometry and loading conditions [8]. The effect of patch shape has also been systematically investigated. Comprehensive experimental and numerical studies have analyzed the influence of CFRP patch shape on fatigue crack growth in aluminum panels under cyclic loading, comparing single-sided circular, square, and rectangular patches. Rectangular patches aligned with the crack direction were most effective, reducing crack growth rates by up to 61.5% and increasing fatigue life by nearly 94% relative to unrepaired specimens. In contrast, circular and square patches were less effective due to limited coverage along the crack path [9]. Advances in numerical modeling, particularly three-dimensional extended finite element methods (XFEM), have facilitated precise simulation of complex crack-front evolution and interfacial debonding in these repaired systems [10].

Campilho et al. conducted combined experimental and numerical investigations of CFRP single-strap repairs under tensile loading, focusing on the effects of overlap length and patch thickness. By implementing a cohesive mixed-mode damage model for ductile adhesives within interface finite elements in ABAQUS^®^, they achieved strong agreement between numerical predictions and experimental results for failure loads, stiffness, and failure modes, thereby demonstrating the robustness of their modeling framework for bonded repairs [11]. Moura et al. employed a combined numerical–experimental approach to examine fatigue crack growth in metal plates repaired with bonded composite patches. Their findings indicated that incorporating adhesive failure improves prediction accuracy and that bonded repairs effectively reduce stress intensity factors and enhance fatigue life [12]. Chen et al. investigated the fatigue behavior of steel plates with multiple holes repaired using bonded CFRP patches through combined experimental and finite element analyses. Their results showed that CFRP repairs significantly reduce ΔK_I_, stabilize crack propagation toward mode-I behavior, and can increase fatigue life by more than three times, while also highlighting the strong influence of hole size and position on repair effectiveness [13]. Madani et al. analyzed stress distributions in notched and cracked aluminum plates repaired with bonded composite patches using finite-element methods. They demonstrated that such repairs reduce stress concentration and SIFs, with double-sided patches providing superior performance by eliminating bending and improving load transfer [14].

Despite extensive research, significant methodological limitations persist, particularly the absence of a unified mechanics-based framework that accounts for the coupled effects of crack size, notch geometry, repair configuration, and adhesive behavior on structural performance. First, most studies rely on isolated, pointwise finite element evaluations [4,15,16]. While these analyses are accurate for specific configurations, they do not provide a continuous geometric-domain representation, which limits the ability to track fracture severity as both crack length and notch diameter vary. Second, research on the mechanical coupling between notches and cracks remains limited [13,17]. Existing models typically treat these discontinuities independently, neglecting the geometric amplification effects arising from their interaction. Third, adhesive evaluation frameworks are generally based on single-metric criteria, such as shear strength or stiffness [18], which do not capture the multidimensional nature of adhesive performance, including ductility, failure reserve, and spatial severity of interfacial stress concentrations. Fourth, current methodologies lack gradient-based analyses for assessing crack-driving stability [9,19]. Without evaluating derivatives of the stress-intensity field, it is not possible to rigorously determine structural sensitivity to geometric perturbations or to identify energetically favorable crack-growth pathways. Fifth, although the benefits of double-sided patch configurations have been recognized [4,20], their contribution to repair symmetry has not been systematically investigated across the full (crack length, notch diameter) design space, leaving their stabilizing effect quantitatively unresolved. Finally, environmental and long-term durability effects are generally examined in isolation [21], rather than being integrated into a comprehensive repair-efficiency framework.

This study introduces an integrated, mechanics-based framework that extends pointwise fracture assessment to a continuous, geometry-aware analysis of bonded composite patch repairs. Stress concentration, stress intensity, fatigue response, and adhesive stresses are represented as smooth, differentiable fields across the crack–notch design space, allowing for evaluation of both magnitude and gradient-driven stability. Adhesive performance is quantified using complementary metrics that encompass load transfer, ductility, failure reserve, and interfacial stress severity. By integrating fracture, fatigue, and adhesive behavior, the framework offers an efficient tool for systematic repair assessment and provides new insights into crack-growth stabilization. Additionally, it enables rapid comparison of various repair configurations without requiring extensive numerical simulations.

## 2. Methodology

### 2.1. Problem Context and Analytical Framework

**Figure 1:**
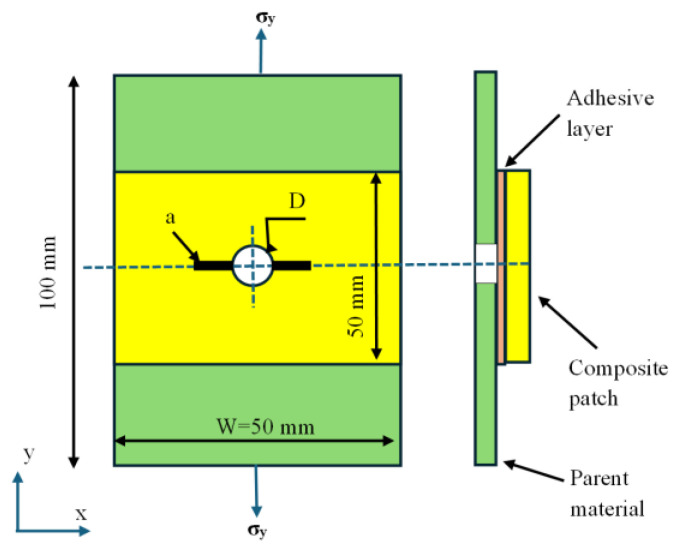
Schematic representation of the bonded composite patch repair system and corresponding loading configuration.

Figure 1 presents the geometric model and loading conditions for a centrally notched, finite-width aluminum plate repaired with a bonded composite patch, following a widely recognized benchmark configuration in the literature [14]. The aluminum plate, measuring 100 mm by 50 mm by 2 mm, contains a centrally located circular notch with diameter *D*. From this notch, a symmetric through-thickness crack of length *a* propagates along the ±*x*-direction. The plate is subjected to a uniform tensile stress (*σ_y_*), resulting in a Mode-I fracture configuration.

Repair of the damaged area is achieved by applying a single- or double-sided rectangular composite patch bonded to the aluminum substrate with a finite-thickness adhesive layer. This arrangement forms a multilayered metal–adhesive–composite assembly. Both the adhesive layer and the composite patch have in-plane dimensions of 50 × 50 mm^2^, providing complete coverage of the notched region. The adhesive and patch thicknesses are 0.125 mm and 1.6 mm, respectively.

### 2.2. Construction of Master Equations

Master equations offer a concise and physically consistent framework for representing the mechanical fields in both unrepaired and repaired configurations. Instead of utilizing discrete finite-element evaluations, this methodology reformulates the mechanical response using continuous, differentiable surfaces defined by the relevant geometric parameters. In this study, two complementary master surfaces are developed: one characterizes the stress-concentration behavior at the notch, while the other represents the mode-I stress-intensity factor at the crack tip. The normalized geometric parameter is defined as

(1) $$\begin{array}{c} x=\frac{D}{W} \end{array}$$

which characterizes the relative severity of the geometric discontinuity with respect to the plate width. This normalization ensures that the resulting formulation is independent of absolute dimensions and instead captures intrinsic geometric scaling. A third-order representation is employed:

(2) $$\begin{array}{c} K_{t}\left(x\right)=a_{0}+a_{1}x+a_{2}x^{2}+a_{3}x^{3} \end{array}$$

This formulation offers adequate flexibility to represent curvature transitions between small and large notch regimes, while mitigating the numerical instabilities and artificial oscillations that often arise with higher-order polynomials. Additionally, its analytical simplicity permits direct differentiation with respect to geometric variables, which is crucial for subsequent sensitivity and gradient-based analyses. Although the notch-based description accounts for initial stress amplification, modeling the evolution of crack-driven behavior necessitates an extended representation that incorporates both crack length and structural scaling effects. To address this, a two-parameter formulation is introduced:

(3) $$\begin{array}{c} x_{1}=\frac{a}{D},x_{2}=\frac{D}{W} \end{array}$$

In this context, the first parameter represents the relative crack growth with respect to the notch size, while the second parameter reflects the global stiffness scaling associated with plate dimensions and the redistribution of stiffness induced by the patch. These parameters were selected due to their physical relevance. The ratio captures the local interaction between crack growth and notch geometry, indicating the transition from notch-dominated to crack-dominated behavior. In contrast, the second parameter accounts for global geometric scaling and finite-width effects, and it is commonly employed in the literature for stress-concentration analysis of notched plates. The stress intensity surface is expressed as

(4) $$\begin{array}{c} K_{I}\left(x_{1},x_{2}\right)=c_{0}+c_{1}x_{1}+c_{2}x_{2}+c_{3}x_{1}^{2}+c_{4}x_{2}^{2}+c_{5}x_{1}x_{2} \end{array}$$

The selection of the polynomial forms in Equations (2) and (4) are guided by both physical considerations and numerical stability. Classical elasticity solutions indicate that stress concentration varies smoothly and monotonically with respect to notch size, without oscillatory behavior. Accordingly, a third-order polynomial is adopted in Equation (2), providing sufficient flexibility to capture curvature transitions while avoiding overfitting and non-physical oscillations. It was observed that this formulation yields high correlation coefficients (R^2^ > 0.98) with low RMSE values, ensuring an optimal balance between accuracy and robustness.

For the stress-intensity formulation in Equation (4), a second-order polynomial surface with interaction terms is employed to capture the coupled effects of crack length and notch size. The inclusion of quadratic and cross terms enables accurate representation of the mild nonlinear interactions between geometric parameters, while maintaining a stable and physically interpretable response. Higher-order terms were examined but did not lead to meaningful improvements in accuracy and were therefore not considered. The selected formulation ensures both robustness and analytical tractability, particularly for subsequent gradient-based analyses.

### 2.3. Repair Efficiency Indices (REIs)

Repair effectiveness is inherently multi-scale, with contributions arising from local notch shielding, crack-tip modification, and global structural redistribution. To capture this behavior, three complementary indices are defined. The local stress-intensity-based efficiency is given by

(5) $$\begin{array}{c} REI_{KI}\left(a,D\right)=1-\frac{K_{I,p}\left(a,D\right)}{K_{I,u}\left(a,D\right)} \end{array}$$

However, pointwise indices alone cannot fully represent the global structural benefit. For this reason, an energy-based metric is introduced:

(6) $$\begin{array}{c} REI_{K_{t}}\left(D/W\right)=1-\frac{K_{t,p}\left(D/W\right)}{K_{t,u}\left(D/W\right)} \end{array}$$

### 2.4. Gradient-Field Characterization of Crack-Driving Forces

The key methodological contribution of the present study is the treatment of the stress-intensity factor as a continuous scalar field defined over a two-dimensional geometric space. Instead of evaluating $K_{I}$ at isolated points, the response is interpreted as a smooth surface in the $\left(a,\;D\right)$ domain, which enables a systematic investigation of sensitivity and crack-driving trends. Within this framework, the gradient of the stress-intensity field is defined as

(7) $$\begin{array}{c} \nabla K_{I}=\left(\frac{\partial K_{I}}{\partial a},\frac{\partial K_{I}}{\partial D}\right) \end{array}$$

and its magnitude is given by

(8) $$\begin{array}{c} |\nabla K_{I}|=\sqrt{{\left(\frac{\partial K_{I}}{\partial a}\right)}^{2}+{\left(\frac{\partial K_{I}}{\partial D}\right)}^{2}} \end{array}$$

The gradient magnitude serves as a direct indicator of the sensitivity of the crack-driving force to geometric variations. Regions characterized by high gradient values correspond to configurations where small changes in crack length or notch size lead to significant variations in the stress intensity factor, indicating unstable or highly sensitive crack-driving conditions. Conversely, regions with low gradient magnitude represent mechanically stable zones, where the response is relatively insensitive to geometric perturbations.

Beyond magnitude, the vector nature of the gradient field provides additional insight into the directional tendencies of crack evolution. By examining the orientation of $\nabla K_{I}$, it becomes possible to infer the dominant driving direction in the geometric parameter space, thereby offering a more comprehensive understanding of crack-growth behavior than conventional pointwise SIF evaluations. To further quantify the local crack-growth tendency, the following derivative is introduced as an incremental crack-driving metric.

(9) $$\begin{array}{c} |\frac{dK_{I}}{da}| \end{array}$$

This quantity reflects the instantaneous sensitivity of the stress intensity factor to crack extension and can be interpreted as an indicator of crack-growth acceleration or deceleration. In contrast to traditional approaches that rely solely on absolute SIF values, this derivative-based measure reveals how different repair configurations influence the evolution of crack-driving forces across early and late propagation stages. Overall, the gradient-field formulation provides a unified and physically interpretable framework for analyzing both the magnitude and the evolution of crack-driving forces, thereby extending conventional fracture mechanics approaches toward a more comprehensive, geometry-aware assessment.

### 2.5. Fatigue-Based Repair Efficiency

To evaluate the influence of repair on fatigue performance, the classical Paris crack-growth law is adopted as the governing framework. The crack propagation rate is expressed as

(10) $$\begin{array}{c} \frac{da}{dN}=C(\Delta K{)}^{m} \end{array}$$

where C and m are material-dependent constants, and ΔK represents the stress-intensity range governing cyclic crack growth. Based on this relation, the total fatigue life is obtained by integrating the crack-growth rate from an initial crack length $a_{0}$ to a critical value $a_{c}$:

(11) $$\begin{array}{c} N=\int_{a_{0}}^{a_{c}}\frac{da}{(\Delta K{)}^{m}} \end{array}$$

This formulation highlights that fatigue life is not governed by a single stress-intensity value, but by the cumulative evolution of the crack-driving force over the entire propagation path. Within the present framework, the effect of repair is introduced through the modification of the stress-intensity field. Since the Paris parameters (C, m) remain unchanged for a given material, the relative fatigue performance between unrepaired and repaired configurations is governed exclusively by differences in the ΔK distribution. Accordingly, a fatigue-based repair efficiency index is defined as

(12) $$\begin{array}{c} F\text{-}REI\left(D\right)=1-\frac{N_{un}}{N_{patched}} \end{array}$$

This definition directly quantifies the life extension achieved by the repair and provides a normalized measure of fatigue improvement. The value of F-REI=0 indicates no improvement, whereas higher values correspond to increasing fatigue-life enhancement due to the patch. Importantly, this formulation decouples the assessment of repair performance from material-specific fatigue parameters. Since both are evaluated using the same Paris law constants, these parameters cancel out in the ratio. Consequently, the fatigue-based repair efficiency index depends only on the relative modification of the stress-intensity factor field.

### 2.6. Adhesive Stress Analysis and Hot-Spot Identification

Adhesive stresses in the overlap region determine both load-transfer efficiency and interfacial durability in bonded patch repairs. Two principal stress components are typically analyzed: shear stress, which primarily facilitates load transfer between adherends, and transverse shear stress, which results from bending mismatch and out-of-plane deformation. These stress components collectively characterize the local interfacial stress state within the adhesive layer. To enable direct comparison across different configurations, the spatial coordinate along the overlap is normalized as

(13) $$\begin{array}{c} \xi =\frac{x-x_{min}}{L_{\mathrm{patch}}} \end{array}$$

thereby mapping all adhesive stress distributions onto a common dimensionless domain. This normalization removes geometric dependencies and enables consistent evaluation of stress distributions across different patch lengths and configurations. Since both stress components contribute simultaneously to interfacial loading, an equivalent adhesive stress is introduced:

(14) $$\begin{array}{c} \tau_{eq}=\sqrt{\tau_{xz}^{2}+\tau_{yz}^{2}} \end{array}$$

This scalar measure provides a unified representation of the state of adhesive stress and facilitates the definition of performance metrics. To quantify adhesive severity, two complementary indices are defined based on $\tau_{eq}$. The first is a peak-based metric,

(15) $$\begin{array}{c} AHI_{max}=max|\tau_{eq}| \end{array}$$

which captures the maximum local stress concentration and directly relates to the risk of interfacial failure initiation. However, peak values alone do not fully describe the structural significance of stress distributions. Therefore, a second metric is introduced:

(16) $$\begin{array}{c} AHI_{\mathrm{int}}=\int_{0}^{1}\tau_{eq}^{2}d\xi \end{array}$$

This metric captures the spatial distribution of adhesive stress severity and the magnitude and extent of stress concentrations, providing a more comprehensive assessment of interfacial loading. However, it should be noted that this measure is solely governed by the interfacial stress field and does not account for the adhesive material’s intrinsic mechanical properties. As a result, while it provides a configuration-level assessment of adhesive stress severity, it cannot be directly used to evaluate the effectiveness of different adhesive systems. To incorporate material-dependent effects, a stiffness-weighted repair index is introduced as

(17) $$\begin{array}{c} A\text{-}REI\left(a,D\right)=REI_{KI}\left(a,D\right)\left(\frac{G}{G_{\mathrm{ref}}}\right)\left(\frac{\tau_{u}}{\tau_{\mathrm{ref}}}\right) \end{array}$$

where G is the adhesive shear modulus and $\tau_{u}$ its shear strength. This formulation integrates fracture mechanics with material properties, facilitating a direct evaluation of the influence of adhesive characteristics on the effectiveness of structural repairs.

## 3. Results

### 3.1. Stress-Concentration Behavior of Repaired and Unrepaired Plates

**Figure 2:**
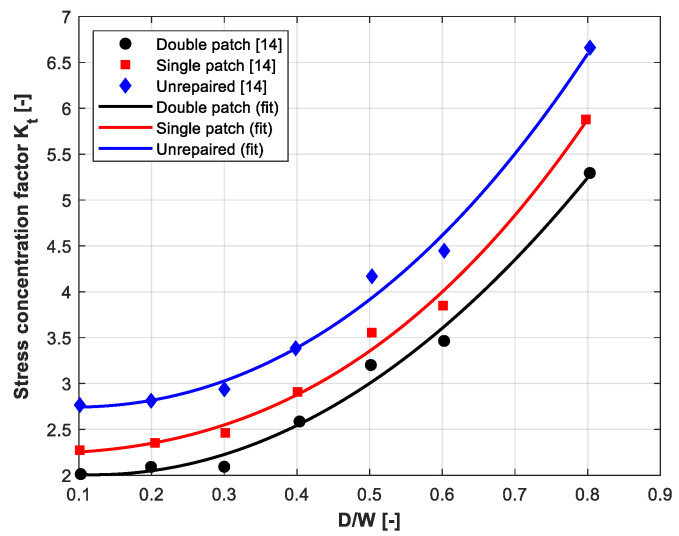
Variation in the stress-concentration factor (*Kₜ*) as a function of the normalized notch size (*D/W*) for unrepaired, single-sided, and double-sided patch configurations.

Figure 2 presents the generalized master curves of the stress-concentration factor *Kₜ(D/W)* for unrepaired, single-sided, and double-sided patched plates. The discrete data points are extracted from the reference study by Madani et al. [14], while the solid curves represent the corresponding master-equation models fitted in the present work. A clear geometric sensitivity is observed: *Kₜ* increases monotonically with *D/W* across all configurations, although the amplification levels differ significantly. The unrepaired plate exhibits the steepest increase in stress concentration, reflecting the classical notch-driven rise in membrane stress. In contrast, both repair configurations significantly reduce *Kₜ*, with the double-sided patch providing the greatest reduction, particularly at higher *D/W* ratios where enhanced membrane stiffness and bending symmetry become more effective [22,23,24].

Table 1 quantitatively confirms these trends by presenting the cubic master-equation coefficients and fitting statistics for each configuration. The high R^2^ values (0.990–0.993) demonstrate that the chosen polynomial form effectively captures the geometric dependence of K_t_. The RMSE values offer additional insight into mechanical stability: the double-patch case shows the lowest error (0.108), indicating a smoother and more predictable response across the entire notch-size range. The single-patch configuration performs similarly well (RMSE ≈ 0.100), although its coefficients suggest a stronger cubic contribution from asymmetric bending effects. Meanwhile, the unrepaired plate has the highest baseline K_t_ values and slightly higher scatter (RMSE ≈ 0.120), consistent with the free notch’s increased sensitivity to geometric variations.

### 3.2. Evolution of the Stress-Intensity Factor $K_{I}$

**Figure 3:**
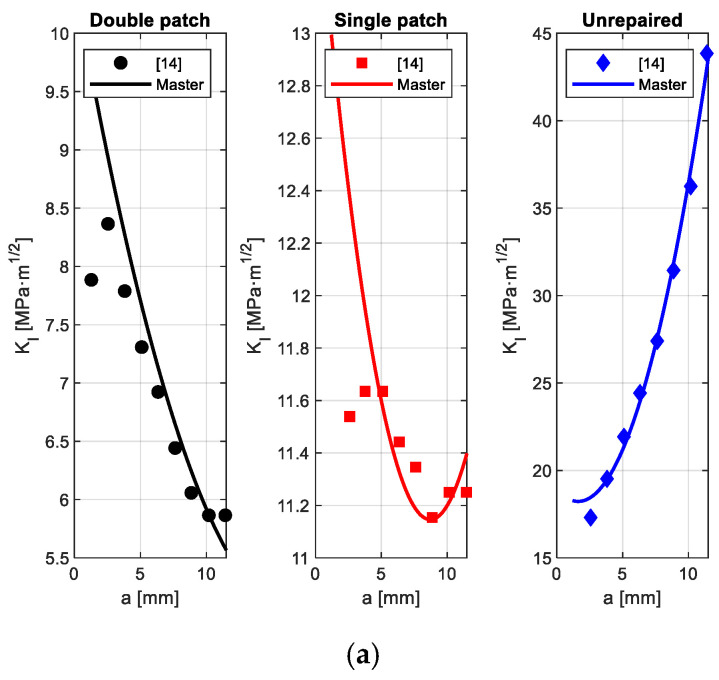

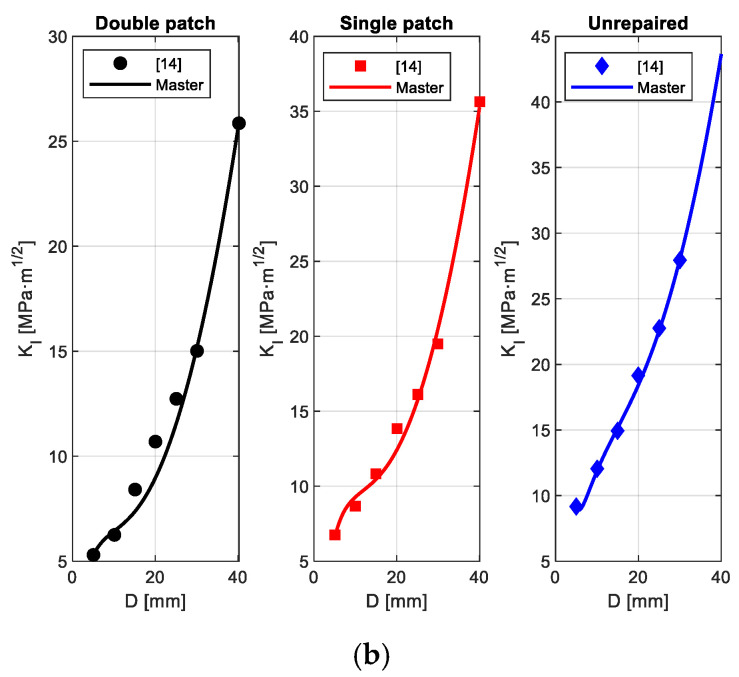
Evolution of the Mode-I stress-intensity factor *K_I_* for unrepaired, single- and double-sided patched plates: (**a**) *K_I_* versus crack length a at *D* = 20 mm; (**b**) *K_I_* versus notch diameter *D* at *a* = 2.5 mm.

Figure 3a illustrates how the Mode-I stress-intensity factor K_I_ varies with crack length a for a fixed notch diameter D = 20 mm. Meanwhile, Figure 3b shows the complementary trend of K_I_ versus notch diameter D for a fixed crack length of a = 2.5 mm. In Figure 3a, the unrepaired plate displays the steepest and most nonlinear increase in K_I_ as the crack length grows, highlighting the typical amplification of crack-tip severity caused by the combined effects of notch-induced membrane stresses and the crack-tip singularity. Conversely, both patch configurations significantly reduce K_I_ across the entire crack-length range, with double-sided reinforcement providing the greatest attenuation. The symmetric patch enhances through-thickness stiffness, diminishes bending curvature, and fosters more uniform membrane load transfer around the crack front [20].

Figure 3b emphasizes the strong dependence of K_I_ on notch diameter D. For the unrepaired setup, even small increases in D lead to significantly larger rises in K_I_, illustrating the inherent geometric coupling of notch–crack systems. Single-patch repairs reduce this sensitivity, though some asymmetry persists due to bending effects from one-sided stiffening. Once again, double-sided patches minimize geometric amplification, yielding the lowest KI values and the shallowest slope with respect to notch size.

Table 2 presents the polynomial coefficients and fitting statistics for the proposed *K_I_ (a,D)* master surface in the unrepaired, single-patch, and double-patch configurations. The large negative values of c_1_ and c_2_ across all configurations confirm that increasing a or D/W significantly worsens fracture severity in an unrepaired plate.

The noticeably smaller RMSE values for patched configurations, especially in the double-patch case, indicate a more stable, smoother, and monotonic mechanical response throughout the entire (a,D) domain. The high coefficients of determination (R^2^ = 0.976–0.996) further confirm that the polynomial surface effectively captures the geometric relationship between crack length and notch size without requiring extensive FE analysis recalculations.

### 3.3. Local Repair Efficiency Based on Stress-Intensity Reduction

**Figure 4:**
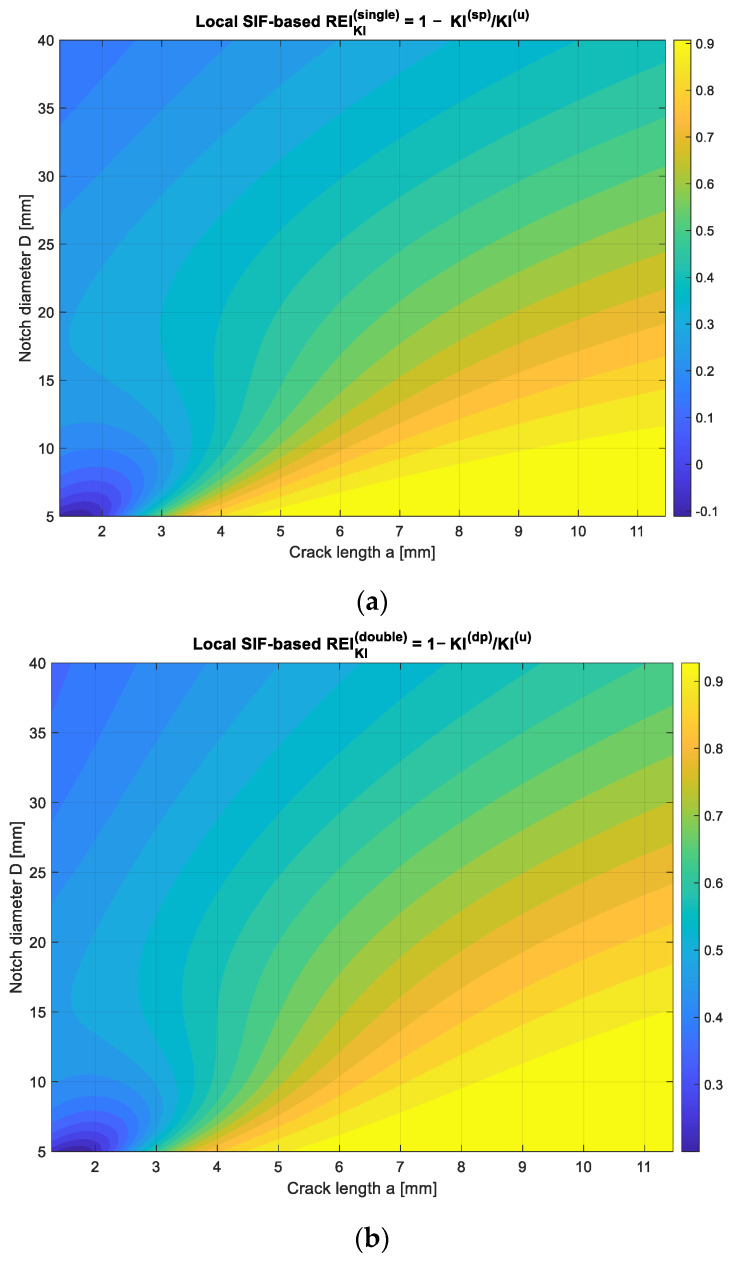
Local repair efficiency index ${\mathrm{REI}}_{K_{I}}(a,D)$ for bonded composite repairs: (**a**) Single-sided patch and (**b**) Double-sided patch.

Figure 4a and Figure 4b illustrate the spatial distribution of the local repair efficiency index for single- and double-sided patch configurations, respectively. This index gauges how effectively a repair diminishes the Mode-I crack-driving force at specific points within the (a,D) design space. In Figure 4a, the single-sided repair results in notable reductions in K_I_, particularly for small cracks and moderate notch sizes. The efficiency gradually decreases with increasing crack length, reflecting the asymmetric stiffness field induced by a one-sided patch [22]. Localized bending distortions restrict the single patch’s capacity to uniformly suppress crack-tip intensity over larger areas of damage.

Figure 4b indicates that the double-sided repair consistently achieves higher repair efficiency across nearly the entire domain. Symmetric reinforcement suppresses both membrane and bending components of the crack-tip stress field, creating broad regions where

(18) $$\begin{array}{c} {\mathrm{REI}}_{K_{I}}\left(a,D\right)>0.6 \end{array}$$

and significantly delay the onset of high-intensity fracture conditions. The smoother contour transitions indicate reduced geometric sensitivity and a more robust redistribution of stresses around the notch–crack system. These spatial observations align with the global energy-based repair efficiency values, which summarize the full-field fracture response into a single scalar metric.

### 3.4. Notch-Based Repair Index Derived from Stress-Concentration Reduction

**Figure 5:**
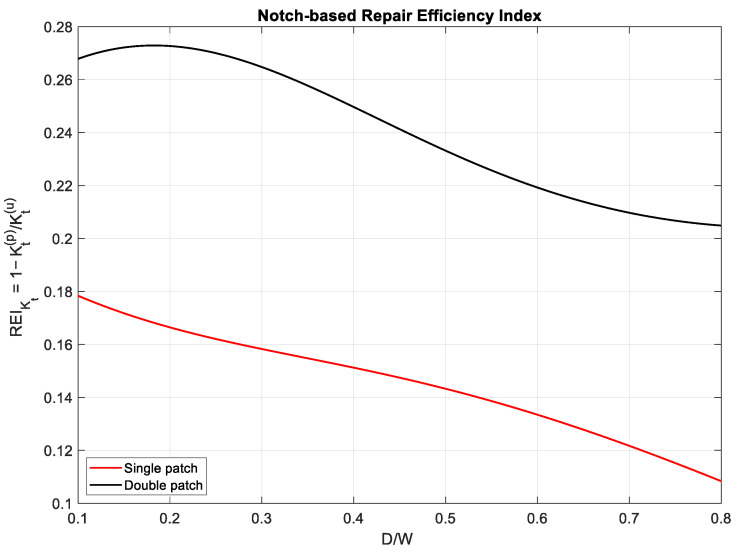
Notch-based repair index ${\mathrm{REI}}_{K_{t}}(D/W)$ for single- and double-sided patched plates.

Figure 5 displays the notch-based repair index, which assesses the bonded patch’s ability to reduce the membrane stress concentration at the notch. Unlike ${\mathrm{REI}}_{K_{I}}$(a,D), which reflects crack-tip behavior, ${\mathrm{REI}}_{K_{t}}$ isolates the notch’s pure contribution to mechanical severity and thus provides a complementary measure of repair effectiveness. The double-sided patch achieves the greatest reduction in K_t_ across all notch sizes, with peak improvements exceeding 40% at D/W ~0.35. This enhanced performance stems from symmetric reinforcement, which increases in-plane stiffness and decreases bending asymmetry at the notch edge, thereby encouraging a smoother stress distribution around the discontinuity. The single-sided patch also significantly reduces the stress concentration factor, especially for smaller notch diameters. However, as D/W increases, the advantages diminish due to increased bending curvature from asymmetric stiffening. This effect reduces the patch’s ability to stabilize membrane stress redistribution, resulting in lower REI_Kt_ values than those of the double-sided patch [9]. A key observation is that both repair options show greater proportional benefits at larger notch sizes, consistent with the expectation that stress concentration increases with notch size. Consequently, even partial mitigation by the patch results in substantial relative improvements.

### 3.5. Gradient-Field Effects on Crack-Driving Forces

**Figure 6:**
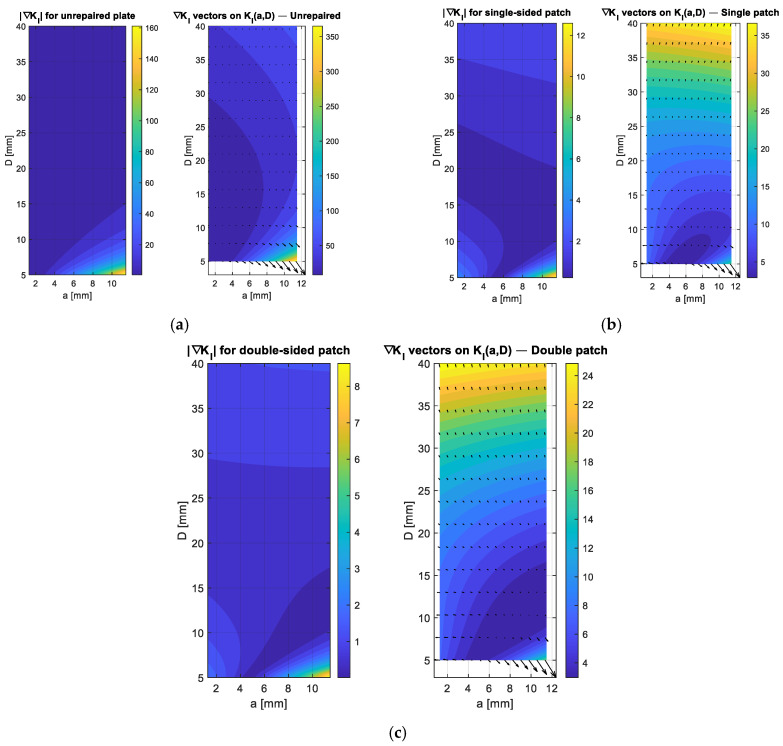
Gradient-field characterization of the crack-driving force $\nabla K_{I}(a,D)$ for unrepaired, single-sided patched, and double-sided patched configurations. (**a**) Unrepaired plate; (**b**) Single-sided patch; (**c**) Double-sided patch.

The evolution of the crack-driving force cannot be fully understood just by the magnitude of the stress-intensity factor K_I_; it also depends heavily on how K_I_ varies with changes in crack length and notch diameter. To capture this behavior, Figure 6a–c illustrates the gradient field $\nabla K_{I}(a,D)=(\partial K_{I}/\partial a,\;\;\partial K_{I}/\partial D)$ in terms of both the distribution of gradient magnitudes and the related vector orientations. In the unrepaired plate, Figure 6a shows that the gradient magnitude is high and concentrated in specific areas, especially where small crack lengths combine with large notch diameters. This steep mechanical landscape indicates that even minor increases in a or D can cause disproportionately large rises in K_I_, leading to a rapid worsening of fracture severity. The vector field points strongly toward increasing crack length, illustrating the inherently unstable nature of the notch–crack interaction. Such behavior reflects the limited tolerance of the unrepaired geometry to small dimensional deviations, which explains the quick structural degradation often observed in experimental studies of notched metallic plates under tension.

Figure 6b demonstrates that using a single-sided patch greatly reduces the gradient magnitude across the entire domain. However, the gradient field becomes noticeably anisotropic. This asymmetry arises because the single patch provides uneven reinforcement, leading to bending curvature and shifting the mechanical response toward the notch-diameter direction. As a result, changes in *D* cause more prominent variations in K_I_ than similar changes in *a*, indicating that while the single-sided repair reduces the geometric sensitivity of the crack-driving force, it does not eliminate it. In regions with intermediate notch sizes, the gradient remains relatively steep, indicating that a single patch cannot uniformly suppress local mechanical amplification across the domain. Figure 6c illustrates the optimal behavior of the double-sided patch setup. The gradient-field magnitude is significantly lower and more evenly distributed than in the other two cases. The vector directions become nearly uniform, indicating a balanced, robust mechanical environment in which geometric changes cause only minor variations in K_I_. The symmetric reinforcement increases both bending and membrane stiffness simultaneously, reducing curvature effects and producing a smoother, less unpredictable fracture pattern. This stabilizing effect enhances resistance to variations in notch and crack length, slows crack growth, and improves overall damage tolerance [18].

### 3.6. Crack-Evolution Path Visualization

**Figure 7:**
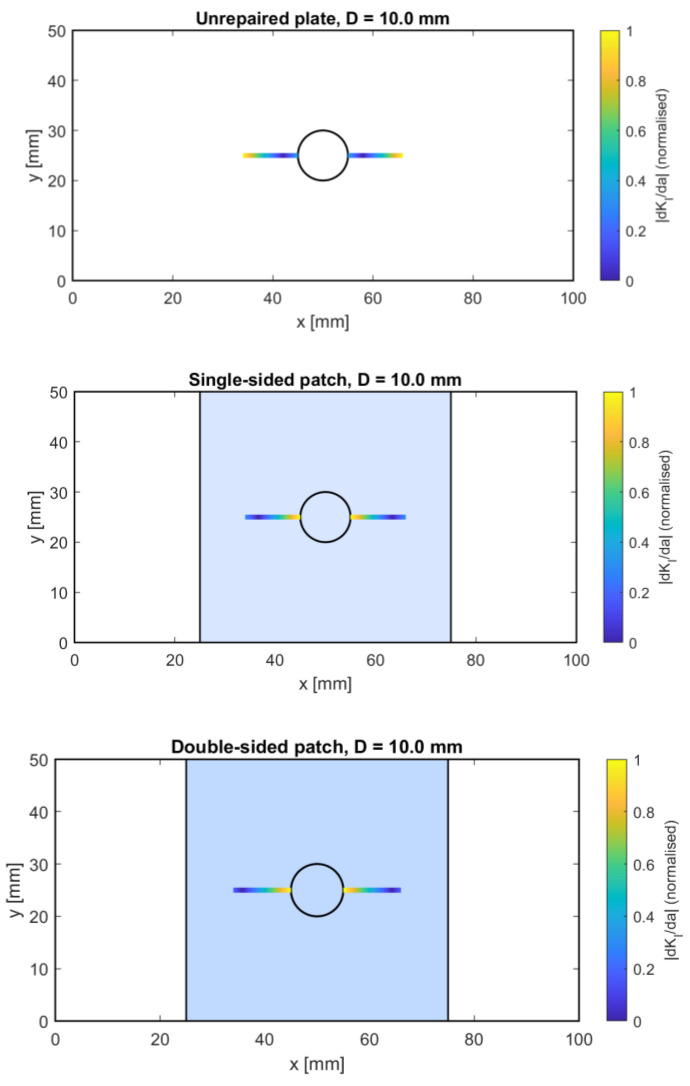
Crack-evolution sensitivity field represented by the normalized derivative $|dK_{I}/da|$ for unrepaired, single-sided patched, and double-sided patched configurations.

Figure 7 provides such a visualization by displaying the normalized sensitivity measure $|dK_{I}/da|$ across the plate for the unrepaired, single-sided-patched, and double-sided-patched configurations. This scalar field represents the local rate at which the crack-driving force increases with incremental crack extension and thus serves as an indicator of the expected crack-acceleration pathways.

In the unrepaired configuration, the spatial distribution of $|dK_{I}/da|$ is dominated by a prominent high-sensitivity band near the notch–crack interface. This region represents an energetically favorable path for crack growth, consistent with the steep gradients identified earlier. The increased sensitivity indicates that even slight crack growth results in significant increases in K_I_, promoting rapid, unstable propagation. This behavior aligns with traditional fracture mechanics expectations for notch-crack interactions in metallic plates and underscores the inherent vulnerability of unrepaired structures.

The single-sided patch significantly reduces sensitivity across the domain, especially near the crack tip. However, the distribution becomes asymmetric due to uneven reinforcement. The reduced stiffness on one side of the plate alters the local curvature and redistributes membrane load, creating preferential pathways in which crack growth remains energetically favored. While the single patch delays crack acceleration, it does not eliminate localized sensitivity peaks, particularly at intermediate crack lengths and notch sizes.

The double-sided patch shows the most significant change in the crack-evolution landscape. The sensitivity field becomes much flatter, with peak $|dK_{I}/da|$ values noticeably lower than in the other two configurations. The reduction in bending asymmetry and the increase in through-thickness stiffness together create a stable mechanical environment in which crack growth does not cause rapid increases in the driving force. Consequently, the double-patched plate exhibits a slower and more controlled crack-evolution path, consistent with delayed crack acceleration and improved overall damage tolerance. The results reinforce the mechanistic understanding developed in the previous sections. Unrepaired plates are susceptible to rapid crack growth due to steep sensitivity gradients; single-sided patches offer partial but directionally biased mitigation; and double-sided patches provide the most stable crack-evolution environment [16].

### 3.7. Effect of Fatigue on Repair Efficiency

**Figure 8:**
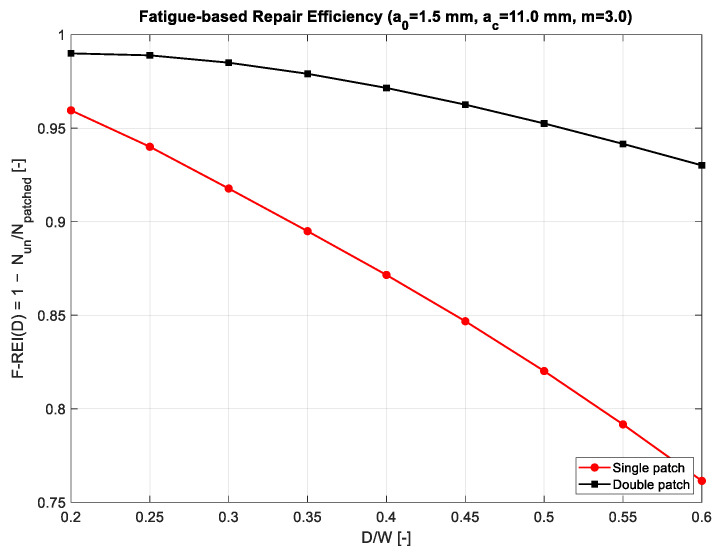
Fatigue-based repair efficiency index F-REI(D) for single- and double-sided patched plates.

Building on the master-equation framework established earlier, Figure 8 shows the fatigue-based repair efficiency index (F-REI). For the unrepaired setup, fatigue life drops sharply as notch diameter increases, due to the rapidly rising stress-intensity amplitude. This pattern aligns with the steep-gradient fields and sensitivity maps shown in Section 3.5 and Section 3.6. Even small geometric changes can lead to significant increases in ΔK_I_, accelerating crack growth and reducing the number of cycles to failure. The single-sided patch significantly improves fatigue life across all notch sizes. This gain comes from reducing both the magnitude of stress and the partial moderation of its gradients. However, the asymmetric bending stiffness of a single patch creates localized sensitivity peaks, limiting the maximum fatigue benefit. As a result, the fatigue-efficiency curve increases moderately but still varies with D, indicating that fatigue performance depends to some extent on geometry. In contrast, the double-sided patch provides the greatest and most consistent increase in fatigue life. The F-REI curve in Figure 8 shows a smooth, steadily rising trend, reaching values of 0.70–0.80 for typical notch sizes. This performance results from several reinforcing factors: the symmetric bending constraint, the significant reduction in ΔK_I_, the minimized gradient magnitudes in the (a,D) domain, and a much flatter crack-evolution sensitivity landscape. These combined effects delay crack growth and result in a fatigue response that is considerably less notch-size-dependent.

Table 3 shows how the fatigue-based repair efficiency index (F-REI) varies with notch size for both single- and double-sided patch configurations. The trends clearly demonstrate a consistent performance hierarchy. For all tested notch diameters, the double-sided patch achieves significantly higher fatigue efficiency, remaining above 0.99 at D/W = 0.20 and above 0.93 even at the largest severity level, D/W = 0.60. This suggests that symmetric reinforcement is very effective in stabilizing the crack-growth driving force throughout the fatigue process, largely preserving the structure’s remaining life despite increased geometric discontinuity. In contrast, the single-sided patch shows a more noticeable decline in F-REI as the notch diameter increases. While it remains highly effective at small notches—reaching 0.96 at D/W = 0.20—its efficiency drops more rapidly, falling to around 0.76 at D/W = 0.60. This sharper deterioration aligns with the bending-induced asymmetry typical of unilateral reinforcement, which increases the crack-driving gradient and reduces fatigue resistance as notch severity grows [12].

### 3.8. Adhesive Stress Master Curves and Hot-Spot Behavior

While the preceding sections focused primarily on crack-tip mechanics and global structural response, the durability and long-term performance of bonded repairs are also strongly influenced by the adhesive layer, where load transfer occurs between the patch and the substrate. 

**Figure 9:**
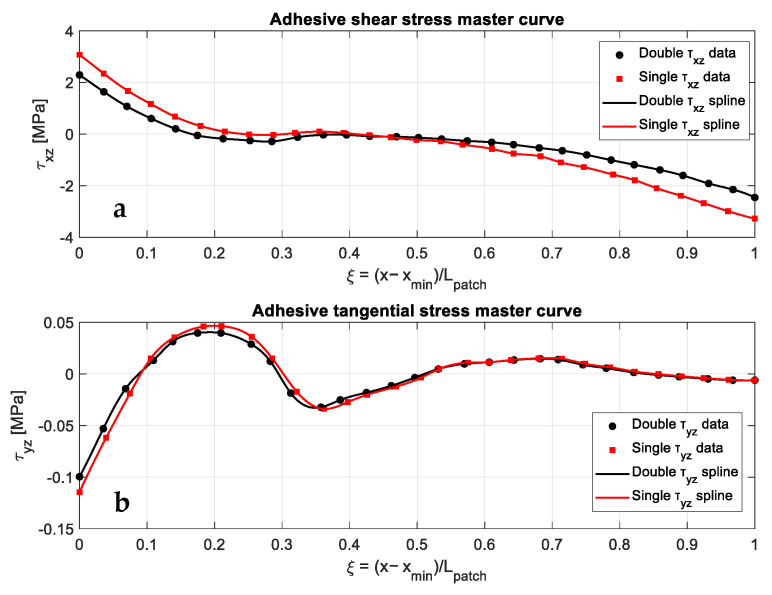
(**a**) Normalized adhesive shear stress master curve $\tau_{xz}(\xi )$ for single- and double-sided repairs. (**b**) Normalized adhesive tangential stress master curve $\tau_{yz}(\xi )$ for single- and double-sided repairs [14].

To examine this aspect, Figure 9a,b presents the normalized adhesive shear and tangential stress distributions along the adhesive interface for single- and double-sided repairs. The discrete data used in these plots were taken from the study by Madani et al. [14] and are shown here as a function of the normalized coordinate to enable direct comparison between the two repair configurations. Figure 9a shows the normalized shear stress distribution along the adhesive interface for both repair configurations. The single-sided repair exhibits higher stress magnitudes and steeper gradients near the overlap ends, whereas the double-sided configuration shows a more gradual and symmetric distribution. 

Figure 9b presents the corresponding tangential stress distribution. Similar trends are observed: the single-sided patch shows larger variations along the overlap, whereas the double-sided configuration yields a more uniform stress profile. These distributions are used in the following sections to quantify the severity of adhesive stress and load-transfer characteristics.

**Figure 10:**
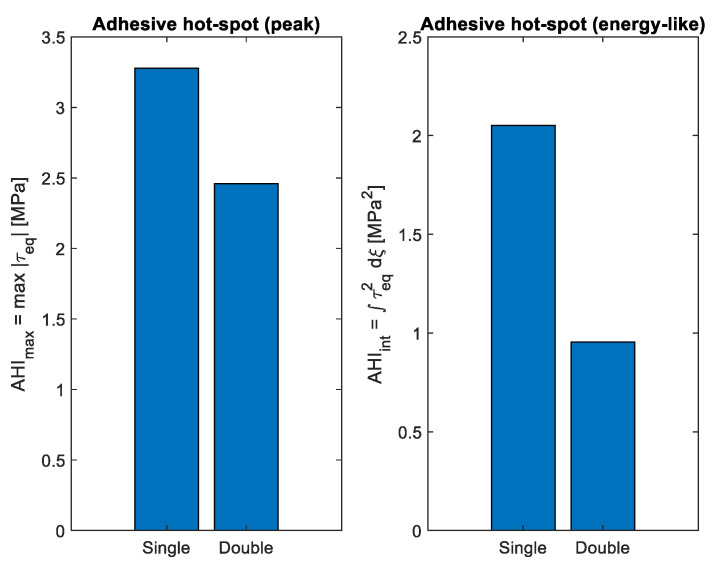
Adhesive hot-spot index comparing single- and double-sided repair configurations.

Figure 10 shows the adhesive hot-spot index, which measures both the peak equivalent adhesive stress and the local stress energy around the interface ends. The hot-spot index clearly distinguishes the behavior of the two repair setups. For the single-sided patch, the index reaches its highest values near the overlap ends, indicating areas of high stress concentration and limited fatigue life. For the double-sided patch, both the size and the extent of the hot-spot region are much smaller, showing a more favorable adhesive environment. This difference has important implications for long-term repair durability: lower hot-spot indices are strongly linked to delayed adhesive damage and longer joint life.

### 3.9. Adhesive Material Selection Using a Unified Repair Efficiency Index

The mechanical effectiveness of a bonded composite repair is governed not only by patch configuration and geometry, but also—critically—by the constitutive properties of the adhesive layer. While the preceding sections quantified repair performance in terms of stress-intensity attenuation and configuration-driven adhesive stress severity, practical design requires the systematic comparison of candidate adhesives with different stiffness and strength characteristics (Table 4).

In this section, the previously defined adhesive-based repair efficiency index (A-REI) (Equation (19)) is employed to evaluate and rank the adhesive systems. By combining fracture-driven efficiency with adhesive stiffness and strength, this index enables a consistent comparison across different materials without introducing additional empirical parameters.

**Figure 11:**
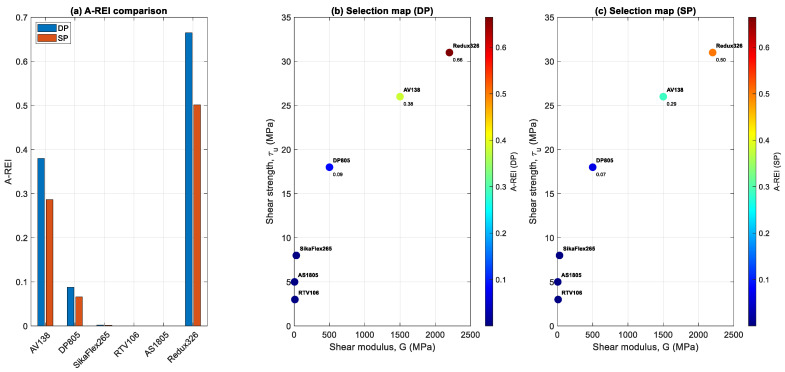
(**a**) A-REI comparison for double- (DP) and single-sided (SP) repairs. (**b**,**c**) Selection map in the G–$\tau_{u}$ space for DP and SP.

Figure 11a presents the adhesive-based repair efficiency index (A-REI) for all candidate adhesive systems under both double-sided (DP) and single-sided (SP) repair configurations. A clear and consistent performance hierarchy is observed across all cases. High-stiffness and high-strength adhesives, particularly Redux326 and AV138, achieve significantly higher A-REI values, indicating superior load transfer capability and enhanced crack-tip shielding. In contrast, low-modulus adhesives such as RTV106 and AS1805 yield A-REI values several orders of magnitude lower, demonstrating negligible contribution to structural repair effectiveness. The results reveal a pronounced separation between adhesive classes. While structural adhesives operate in the range of $10^{-1}$, flexible adhesives drop to $10^{-3}$–$10^{-4}$, corresponding to differences approaching three orders of magnitude. This clearly indicates that adhesive selection is not a secondary parameter but a dominant design variable governing repair performance. Additionally, for all adhesive systems, the double-sided configuration consistently produces higher A-REI values than the single-sided case. This trend confirms that repair symmetry acts as a global amplification mechanism, enhancing the effectiveness of material properties by improving load redistribution and reducing bending-induced inefficiencies. Figure 11b displays the adhesive selection map in the G–$\tau_{u}$ space for double-sided repairs, with the color scale indicating the magnitude of A-REI. There is a strong monotonic relationship between adhesive mechanical properties and repair effectiveness. Adhesives in the upper-right region of the map, defined by high shear modulus and high shear strength, correspond to the highest A-REI values. This distribution demonstrates that both stiffness and strength contribute multiplicatively to repair performance, as reflected in the A-REI formulation. Increased stiffness enhances load transfer and crack-tip shielding, whereas higher strength improves resistance to interfacial failure. The combined effect results in a steep performance gradient across the material space, distinctly separating high-performance structural adhesives from low-performance flexible systems. The clustering of low-performance adhesives near the origin indicates that insufficient stiffness cannot be compensated by moderate strength levels. This finding underscores a critical design insight: stiffness is essential for effective bonded repair, and strength contributes meaningfully only when sufficient load transfer is established.

Figure 11c presents the corresponding selection map for single-sided repairs. Although the overall distribution and ranking are consistent with the double-sided case, all A-REI values are systematically reduced. This result indicates that the influence of adhesive properties is maintained, but their effectiveness is constrained by the asymmetric structural configuration. The reduction in A-REI across all materials reflects bending-induced inefficiencies in load transfer inherent to single-sided repairs. Even high-performance adhesives such as Redux326 demonstrate reduced effectiveness compared to the double-sided configuration. Nevertheless, the relative positioning of adhesives remains unchanged, confirming that material selection and structural configuration function as decoupled yet complementary design factors. From a design perspective, these results indicate that although adhesive optimization can enhance repair performance, it cannot fully compensate for structural asymmetry. Maximum efficiency is achieved only when optimal material selection is combined with a symmetric repair configuration.

## 4. Conclusions

This study presents a unified mechanics-based framework for evaluating bonded composite patch repairs by transforming discrete fracture, fatigue, and adhesive responses into continuous master equations over the $\left(a–D\right)$ design space. The following conclusions were obtained:

- The proposed framework provides a continuous and physically consistent representation of notch–crack behavior, showing high agreement with reference solutions and enabling reliable prediction without repeated finite-element analyses.
- The fatigue response is strongly dependent on geometric severity. As the notch ratio increases from D/W 0.20 to 0.60, the efficiency of single-sided repair decreases significantly (≈0.96→0.76), whereas double-sided repair maintains consistently higher performance (≈0.99→0.93).
- In terms of stress concentration, double-sided repair provides the most effective mitigation, with reductions exceeding 40% at intermediate notch ratios, highlighting the role of bending symmetry in suppressing peak stresses.
- Adhesive selection is governed by stiffness and strength, as captured by the A-REI metric. Structural adhesives (e.g., Redux326) achieve significantly higher efficiency, while compliant adhesives exhibit negligible contribution to repair effectiveness.
- Overall, repair symmetry controls the magnitude of improvement, while adhesive properties determine performance ranking, providing a clear and practical basis for design and material selection.

## Figures and Tables

**Table 1:** Polynomial coefficients for K_t_ (D/W) master curve.

Configuration	a_0_	a_1_	a_2_	a_3_	RMSE	R^2^
Double patch	2.10355	−1.62280	6.68026	0.31837	0.10804	0.99016
Single patch	2.24551	−0.27810	3.26186	3.45618	0.09955	0.99293
Unrepaired	2.79428	−1.09394	5.61073	2.11885	0.11981	0.99137

**Table 2:** Polynomial coefficients for the *K_I_* (*a*,*D*) master curve.

Configuration	c_0_	c_1_	c_2_	c_3_	c_4_	c_5_	RMSE	R^2^
Double patch	13.1060	−15.3072	−29.711	7.662	57.71846	5.992	0.7701	0.9764
Single patch	21.2963	−31.9673	−54.979	13.067	89.54079	51.425	0.5426	0.9931
Unrepaired	29.391	−102.8287	−64.288	103.753	95.24384	214.886	0.5568	0.9962

**Table 3:** Fatigue-based Repair Efficiency Index F-REI(D/W) for Single- and Double-Sided Repairs.

D	D/W	F-REI (Single Patch)	F-REI (Double Patch)
10.0	0.20	0.9595	0.9900
12.5	0.25	0.9401	0.9889
15.0	0.30	0.9177	0.9850
17.5	0.35	0.8949	0.9791
20.0	0.40	0.8715	0.9715
22.5	0.45	0.8468	0.9626
25.0	0.50	0.8202	0.9525
27.5	0.55	0.7916	0.9416
30.0	0.60	0.7615	0.9301

**Table 4:** Mechanical properties of the candidate adhesives used in this study.

Adhesive Name	G (MPa)	τ_u_ (MPa)	Ultimate Shear Strain γ (-)
AV138	1600	26	1.2
DP805	550	18	0.8
SikaFlex265	0.7	7	1.5
RTV106	0.5	3	2.2
AS1805	0.4	4	1.8
Redux326	2100	31	0.9

## Data Availability

The data presented in this study are available from the corresponding author upon reasonable request. The MATLAB R2024b code developed for data processing, master-curve construction, and repair-efficiency analysis is available upon request. The data are not publicly available because they are directly associated with an ongoing research framework.

## Abbreviations

The following abbreviations are used in this manuscript:

A-REI	Adhesive-based Repair Efficiency Index
CFRP	Carbon Fiber Reinforced Polymer
FE	Finite Element
F-REI	Fatigue-based Repair Efficiency Index
K_I_	Mode-I Stress Intensity Factor
K_t_	Stress Concentration Factor
R^2^	Coefficient of Determination
REI	Repair Efficiency Index
REIK_I_	Stress-intensity-based Repair Efficiency Index
RMSE	Root Mean Square Error
SIF	Stress Intensity Factor
SIFs	Stress Intensity Factors
XFEM	eXtended Finite Element Method
a	Crack length
D	Notch diameter
W	Plate width
x_1_	Normalized crack length (a/D)
x_2_	Normalized notch size (D/W)
ξ	Normalized coordinate along adhesive overlap
σᵧ	Applied far-field tensile stress
ΔK	Stress intensity factor range
N	Fatigue life (number of cycles)
a_0_	Initial crack length
a_c_	Critical crack length
C	Paris law coefficient
m	Paris law exponent
∇KI	Gradient of stress intensity factor
|∇K_I_|	Magnitude of gradient
dK_I_/da	Crack-driving sensitivity
τ_xz_	Adhesive shear stress
τ_yz_	Adhesive transverse shear stress
τ_eq_	Equivalent adhesive stress
AHI_max_	Maximum adhesive stress index
AHI_int_	Integrated adhesive stress index
G	Adhesive shear modulus
G_ref_	Reference shear modulus
τ_u_	Adhesive shear strength
τ_ref_	Reference shear strength
a_0_, a_1_, a_2_, a_3_	Coefficients of stress concentration polynomial
c_0_–c_5_	Coefficients of stress intensity polynomial
